# A Framework for Assessing Global Change Risks to Forest Carbon Stocks in the United States

**DOI:** 10.1371/journal.pone.0073222

**Published:** 2013-09-10

**Authors:** Christopher W. Woodall, Grant M. Domke, Karin L. Riley, Christopher M. Oswalt, Susan J. Crocker, Gary W. Yohe

**Affiliations:** 1 US Department of Agriculture, Forest Service, Northern Research Station, St. Paul, Minnesota, United States of America; 2 College of Forestry and Conservation, University of Montana, Missoula, Montana, United States of America; 3 US Department of Agriculture, Forest Service, Southern Research Station, Knoxville, Tennessee, United States of America; 4 Wesleyan University, Middletown, Connecticut, United States of America; DOE Pacific Northwest National Laboratory, United States of America

## Abstract

Among terrestrial environments, forests are not only the largest long-term sink of atmospheric carbon (C), but are also susceptible to global change themselves, with potential consequences including alterations of C cycles and potential C emission. To inform global change risk assessment of forest C across large spatial/temporal scales, this study constructed and evaluated a basic risk framework which combined the magnitude of C stocks and their associated probability of stock change in the context of global change across the US. For the purposes of this analysis, forest C was divided into five pools, two live (aboveground and belowground biomass) and three dead (dead wood, soil organic matter, and forest floor) with a risk framework parameterized using the US's national greenhouse gas inventory and associated forest inventory data across current and projected future Köppen-Geiger climate zones (A1F1 scenario). Results suggest that an initial forest C risk matrix may be constructed to focus attention on short- and long-term risks to forest C stocks (as opposed to implementation in decision making) using inventory-based estimates of total stocks and associated estimates of variability (i.e., coefficient of variation) among climate zones. The empirical parameterization of such a risk matrix highlighted numerous knowledge gaps: 1) robust measures of the likelihood of forest C stock change under climate change scenarios, 2) projections of forest C stocks given unforeseen socioeconomic conditions (i.e., land-use change), and 3) appropriate social responses to global change events for which there is no contemporary climate/disturbance analog (e.g., severe droughts in the Lake States). Coupling these current technical/social limits of developing a risk matrix to the biological processes of forest ecosystems (i.e., disturbance events and interaction among diverse forest C pools, potential positive feedbacks, and forest resiliency/recovery) suggests an operational forest C risk matrix remains elusive.

## Introduction

**Figure 1:**
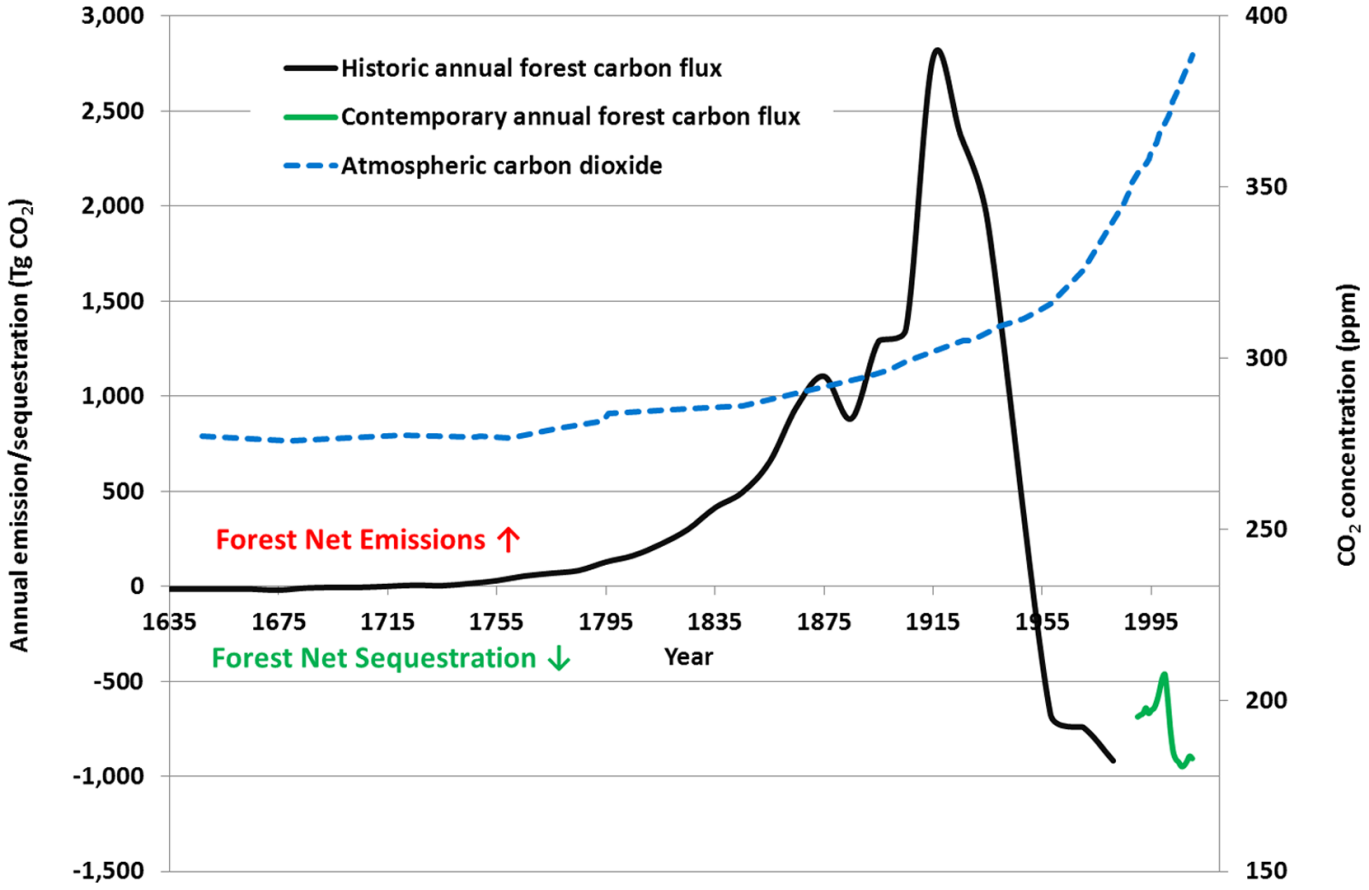
Historic annual rates of forest ecosystem and harvested wood product carbon dioxide net emissions/sequestration in US forests (black line: Birdsey et al. [7], green line: EPA [10]) and global atmospheric CO_2_ concentration (Etheridge et al. [8], ESRL [9]), 1635 to 2010.

As the current carbon (C) stocks of forests in the United States (US) store an amount of C approximately equal to 25 years' worth of US fossil fuel CO_2_ emissions at their current rate [1], the status and fate of these C stocks in the face of global change is an area of emerging concern [2], [3], [4], [5]. The loss and subsequent partial recovery of forests in the US following the exploitive harvests and land-use conversion of the late 19^th^ and early 20^th^ century are a past event that can frame current discussions regarding managing/monitoring forest C stocks across large-scales [6]. The relatively rapid change in the status of forests in the US – from a steady state of minimal CO_2_ emission/sequestration to major CO_2_ emitter – during this time period (Fig 1) [7] may offer a cautionary tale of how quickly the source/sink status of large-scale forest C stocks can change. Despite this transcontinental degradation of forests in the US and concomitant emission of CO_2_, global atmospheric CO_2_ concentrations only slightly increased above pre-industrial levels during this period [8], [9].

The advent of modern forestry in the US, conservation movements, and urbanization of the populace rapidly shifted the status of forests in the US so that they once again provided a net sequestration of C during the mid-20^th^ century to the present day [7], [10]. While it was a direct human disturbance (e.g., logging and land-use conversion) that precipitated the last US forest status change to a net emission source, could climate change (CC) and its potential to increase the probability of large-scale disturbance events and/or alterations to forest C cycles [11], [12], [13], [14] result in a similar shift in sink/source status now that global CO_2_ atmospheric concentrations approaching 400 ppm [9]? There is growing evidence that large-scale natural disturbances contribute to substantial C emissions from forests on an annual basis [3], [15]. Given forests are the largest terrestrial C sink on earth [16] much of this C may be at risk. One of the most critical future consequences of CC on forest C stocks is the potential change in their source/sink status (i.e., net annual sequestration to net annual emission). Although forests currently sequester more C than they emit on an annual basis globally [16], the ability of forests to continue this trend in the future may be limited [7], [17].

Developing a conceptual framework for assessing CC risks to forest ecosystem C stocks may enable efficient allocation of efforts to monitor and mitigate CC effects while informing future research into refined risk probabilities. In general, risk has been organized around two components, likelihood and consequence(s) (i.e., expectation of random variable) [18]. The exact metrics used to assess risk vary based on available data and information deemed important by stakeholders [19]. These metrics can be organized using a matrix where the magnitude of consequences is plotted against the relative likelihood of the event occurring [20]. Within the matrix, a series of grids can be developed to represent functional levels of risk which translate into monitoring strategies aimed at managing the risk [21], [22]. This approach has been adopted by Iverson et al. [20] to evaluate the risk of CC on forested habitats. The creation of the risk matrices was not intended for use in decision making; rather the matrices were designed to focus the evaluation of risk in terms of likelihood and consequence [21], [22]. Although Iverson et al.'s [20] focus was on risk of habitat change for tree species, the opportunity exists to extend the methodology to other forest ecosystem attributes and services (e.g., water or C). In the context of CC and forest C dynamics, risk may be conceptualized as the magnitude of C stock change due to CC and/or CC-induced natural disturbances multiplied by the associated probability that C stocks might change due to said events. Given the need to broadly assess global change risks to forest C stocks, the goal of this study was to develop and evaluate a general framework (i.e., risk matrix) for forest C stocks in the US using estimates from the National Greenhouse Gas Inventory (NGHGI) in the US and associated forest inventory data to parameterize the initial matrix across broad climate zones.

## Methods

The basic premise of this study's proposed forest C risk matrix is that risk is a combination [20], [22] of 1) the likelihood of a forest C pool's emission and 2) the consequence of such an emission. Within the risk framework, the consequence is equated with the relative size (i.e., mass) of each forest C pool and plotted along the y-axis. The consequences of a forest C pool shifting from a sink to a source is postulated as being closely related to its population estimate over a large region of interest, in this case, the conterminous US. The likelihood of such a shift is equated with each forest C pool's variability across climate zones of the US – higher variability equates to a greater likelihood of change. In this analysis, total forest ecosystem C was apportioned into five pools (live above- and belowground biomass, dead wood, soil organic C, and forest floor) as broadly delineated by the United Nations Framework Convention on Climate Change [10]. The combination of each C pool's consequence and likelihood arrays itself within a matrix of societal responses [20] to CC including combinations of monitoring, mitigation, and adaptation. Given the complexities of human-forest-climate interactions, the size and arrangement of societal responses within the risk framework would vary with scale, geographic area, socioeconomic drivers and constraints, forest types, and existing monitoring infrastructure, among other factors.

The magnitude of C stocks (i.e., consequence) in each pool was estimated from the US Department of Agriculture's Forest Inventory and Analysis (FIA) program [23], which informs the NGHGI of the US [10]. For the purposes of FIA's inventory, forest is defined as at least 36.6 m wide and 0.4 ha in size with at least 10 percent cover by live trees. The FIA program employs a three phase inventory. Phase one is a variance reduction process where satellite imagery is used to assign individual field plots to strata (e.g., forest canopy cover classes). FIA's plot network contains over 125,000 plots which are systematically distributed approximately every 2,428 ha across the conterminous US. During the second phase of the inventory, if a sample point falls in a forested area then field crew visit the plot. Each forested plot is comprised of a series of smaller sub-plots where tree- and site-level attributes – such as diameter and tree height – are measured at regular temporal intervals [23]. During the third phase of the inventory, a subset of phase two plots are measured for additional variables related to forest health attributes (e.g., downed woody materials, understory vegetation, and soils). The FIA program does not directly measure forest C stocks. Instead, a combination of empirically derived C estimates (e.g., standing live and dead trees) [24] and models (e.g., forest floor C stocks related to stand age and forest type) are used to estimate forest C stocks [10]. Estimates of C stocks by pool were developed for 2010 using FIA's current inventory. For illustrative purposes, current C stocks were projected to the year 2100 using annual estimates of forest C stocks over the UNFCCC reporting period (1990-present) [10] and simple linear regression techniques by pool. Although it is not expected that forests will continue to sequester C at current rates to the year 2100 (especially for soil organic C), this parsimonious approach facilitates interim evaluation of the risk matrix.

To estimate the likelihood of forest C stock changes, current (1976–2000; referred to hereafter as 2010) and future projected Köppen-Geiger climate zones (2076–2100; referred to hereafter as 2100) from Rubel and Kotek [25] were used. The A1F1 emission scenario and associated projected climate zones (2100) were selected from Rubel and Kotek [25] as it demonstrated the largest future climate shift for illustration purposes in this study. The FIA program's plot network was overlaid with current (2010) and A1F1 Köppen-Geiger climate zones (2100) [25] for estimating the coefficient of variation of the median C stock densities among the various climates by pool for each year (2010 and 2100). In order to project coefficients of variation in the year 2100, a basic imputation approach was employed. If a FIA plot's current climate zone changed between 2010 and 2100 [25], then the plot was assigned the median C density of that projected future climate using that climate's current median C density by pool. Coefficients of variation were then re-calculated using combined data from current (i.e., no change in climate zone) and imputed (i.e., change in climate zone) values. For example, if half of FIA's forested plots experienced a shift in climate zones from 2010 to 2100, then half of the plot-level 2100 C densities would be based on 2010 C empirical estimates while half of the plots would be assigned the median C density associated with the new climate zone in which the plots are located. Hence, future forest C stock variability would be based on knowledge of the current distribution of C stock densities among climates assuming that current climates would still exist in 2100 but at different locations (e.g., arid zone with different spatial extent). For more information on: 1) population estimation procedures used by the FIA program please refer to Bechtold and Patterson [23], 2) forest C pool models and inventory data specific to 2010 please refer to the EPA [10], and 3) current and projected Köppen-Geiger climate classifications please see Rubel and Kottek [25].

## Results

Current forest C stocks were estimated for the year 2010 by pool. Soil organic C was the largest stock at 17,572 Tg and dead wood the smallest at 2,627 Tg (Table 1). Associated univariate statistics suggest substantial variability in plot-level estimates of forest C density (Mg⋅ha^−1^) by pool with the standard deviation exceeding the mean for live aboveground, live belowground, and dead wood in 2010.

**Table 1:** Estimates of total forest ecosystem C stocks (Tg), mean and associated standard deviation (SD) of carbon density, and associated univariate statistics (Q1: first quartile, median, Q3: third quartile; Mg⋅ha^−1^), across the national forest inventory by carbon pool in the US, 2010.

Carbon pool	Total stocks	Mean	SD	Q1	Median	Q3
Live AG	14,541	38.73	40.57	10.47	27.35	54.76
Live BG	2,876	8.02	8.66	2.14	5.69	11.26
Dead Wood	2,627	8.43	10.70	3.71	6.17	9.61
Forest Floor	4,941	16.08	11.85	7.20	10.20	24.20
SOC	17,572	71.38	48.25	41.7	53.1	94.80

Note: estimates do not include Hawaii, Alaska, or trees on non-forest land (e.g., agricultural trees and urban parks).

* AG = aboveground, BG = belowground, dead = standing and downed dead wood, SOC = soil organic carbon.

As an initial appraisal of empirical variation in C stocks across the various climate zones of the US in 2010, the coefficients of variation (percent) of median C densities (based on individual plot-level estimates of C stocks) were calculated by pool and climate zone across the US and are ordered as: soil organic C (70.9), dead wood (55.2), forest floor (53.4), live belowground (44.6), and live aboveground (43.1) (Table 2). The highest median C density (Mg⋅ha^−1^) by pool across climates in 2010 was live aboveground (33.83), live belowground (7.95), dead wood (11.36), forest floor (33.11), and soil organic C (158.01) in the warm temperate (summer dry), warm temperate (summer dry), warm temperate (summer dry), snow (summer dry), and equatorial, respectively.

**Table 2:** Estimates of median forest carbon density (Mg⋅ha^−1^) by carbon pool and Köppen-Geiger Climate Classifications [25] with coefficients of variation (CV) determined across climates for each carbon pool, 2010.

Pool	Climate Classification						CV (%)
	Equatorial	Arid	Warm Temperate, fully humid	Warm Temperate, summer dry	Snow, fully humid	Snow, summer dry	
	median carbon density (Mg⋅ha^−1^)						
Live AG	14.02	8.05	29.25	33.83	28.56	29.63	43.1
Live BG	2.85	1.68	6.00	7.35	5.94	6.60	44.6
Dead Wood	3.95	1.46	5.85	11.36	6.78	9.02	55.2
Forest Floor	7.26	21.09	7.84	28.14	20.28	33.11	53.4
SOC	158.01	24.12	45.95	49.80	94.76	44.12	70.9

* AG = aboveground, BG = belowground, dead = standing and downed dead wood, SOC = soil organic carbon.

For illustrative purposes, C stocks and associated coefficients of variation among climate zones were projected to the year 2100 (Table 3). Projected C stocks increased in all forest C pools over the 90 year period (2010–2100), with the largest increases occurring in live aboveground biomass (49 percent; 7,116 Tg) and belowground biomass (49 percent; 1,406 Tg) followed by dead wood (25 percent; 671 Tg), soil organic C (8 percent; 1,331 Tg), and forest floor (7 percent; 334 Tg) (Table 3). The projected coefficients of variation of C pools among climate zones in 2100 increased for all pools. The largest increase (absolute change in coefficient percentage, percent) was for forest floor (4.2) followed sequentially by soil organic C (2.1), live belowground (0.5), dead wood (0.4), and live aboveground (0.2).

**Table 3:** Linear projections (year 2100) of total forest carbon stocks (Tg) based on contemporary baselines by carbon pool and projections of future coefficients of variation (CV) of carbon pools determined across A1F1 Köppen-Geiger Climate Classifications (years 2076–2100) [25].

Pool	Total Carbon Stock (Tg)	CV (%)
Live AG	21,657	43.3
Live BG	4,282	45.1
Dead Wood	3,298	55.6
Forest Floor	5,275	57.6
SOC	18,903	73.0

* AG = aboveground, BG = belowground, dead wood = standing and downed dead wood, SOC = soil organic carbon.

**Figure 2:**
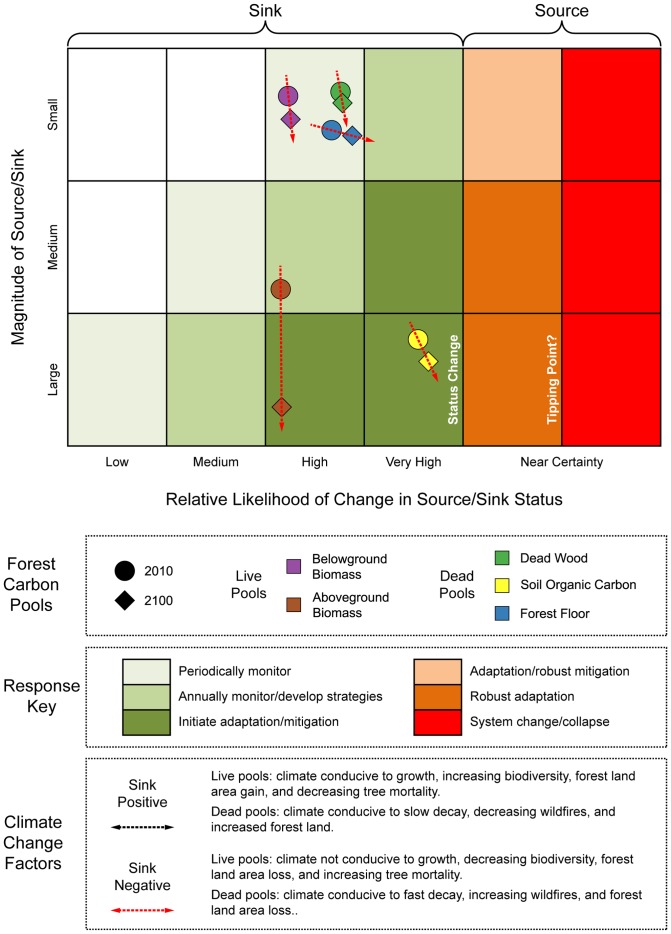
Climate change risk matrix for forest ecosystem carbon pools in the US. Likelihood of change in carbon stocks is based on the coefficient of variation of median forest carbon stock densities among Köppen-Geiger climate regions (i.e., x-axis) based on the national forest inventory plot network. Size of carbon stocks are based on the US National Greenhouse Gas Inventory (i.e., y-axis). Societal response (e.g., immediate adaptive response or periodic monitoring) to climate change events depends on the size and relative likelihood of change in stocks. Year 2100 projections are based on linear extrapolations of current carbon stocks and imputing current median carbon pool densities by climate region to projected future climate regions for calculation of coefficients of variation. The soil organic carbon pool exhibits the highest variability among climate regions and therefore may be most affected by climate change or climate change induced disturbance events. In contrast, the dead wood pool has a relatively small stock with low variability among climate regions. Explicit climate change effects are not incorporated into this matrix as they represent a number of complex feedbacks both between stocks (e.g., live aboveground biomass transitioning to the dead wood pool) and the atmosphere (e.g., forest floor decay).

The estimates of current US forest C stocks by pool in conjunction with coefficients of variation among climate zones were used to array the forest C pools in a risk matrix (Fig 2). Pools were arrayed within the risk matrix as a combination of their associated stock size (i.e., magnitude of C sink/source) and C density variability among climates (i.e., likelihood of change under future CC). Because both the stocks and coefficients of variation were projected to the year 2100, the combination of population estimates (2010 and 2100) could be plotted within the risk matrix (i.e., general societal response key) which allowed for broad assessment of potential trends in forest C stocks and discussion of monitoring strategies. Soil organic C had the largest stock estimate in 2010 combined with a highest level of variation across climate zones and thus fell within the “initiate adaptation/mitigation” response category (Fig 2). As the remainder of pools had coefficients of variation within the same narrow range (43.1–55.2 percent), their assignment to response categories was largely dependent on their current stock size (i.e., mass). As the live aboveground pool had the second largest stock it was assigned to the “annually monitor/develop strategies” response category. The three pools of live belowground, forest floor, and dead wood had relatively small estimates of C stocks combined with moderate to low levels of variability among climates, thus their response category assignments were limited to periodic monitoring activities.

For the purpose of discussion, C stocks and associated variability among climates was projected to the year 2100 and arrayed in the risk matrix (Fig 2). All pools moved in varying degrees towards more proactive response categories (i.e., from periodically monitor to robust mitigation). For some pools, such as the forest floor and soil organic C, their movement towards a potential source was largely due to the increase in their coefficients of variability among climate zones in 2100. For other pools, such as live aboveground, there was minimal increase in risk of emission due to increases in their respective stock sizes.

## Discussion

Empirically derived estimates (e.g., population totals and associated variability across climates) of forest C pools across the US may be used to develop an initial CC risk matrix. This risk matrix is not intended to be used to develop local strategies to mitigate CC and disturbances to forest C cycles and stocks; however, it may provide a common framework for discussing risks across large spatial/temporal scales. As the risk matrix attempts to mesh empirical estimates with societal response, a thorough understanding of its limits, implications, and potential refinements is needed.

As the basic premise of the risk matrix is that the risk of C pool status change (i.e., C source or sink) relates to 1) the likelihood (i.e., coefficients of variation for each individual forest pool's median C density across climate zones) and 2) the consequence of such a shift (i.e., forest C pools' current stock). Coefficients of variation were used as an initial metric of change likelihood as it facilitated comparison across diverse forest C pools and has been used in risk assessments in the biological sciences [26] and finance [27]. If indeed CC occurred such that a forest experienced a climate shift from “temperate” to “equatorial,” then the contemporary range in variation in C densities between those climates may indicate likelihood of C emission. Because societal responses can only be conjectured in this study, the proposed C risk framework suggests C source/sink status change and a tipping point where forest C emissions may exacerbate CC impacts and positive feedbacks (e.g., boreal forest heterotrophic respiration, [28]) as metrics critical to society. In practice, the size and arrangement of societal responses must align with socioeconomic drivers and constraints, the forest types and attributes that may be at risk, and existing monitoring infrastructure, among other factors.

In a manner similar to previous work [20], the risk matrix in this study is not intended to be used in decision making; rather it is designed to focus attention on the short- and long-term risks to forest C stocks. This is particularly true given the large amount of uncertainty associated with C stocks and stock changes associated with CC and intensified forest disturbances. Time is not explicitly part of the risk matrix to allow consideration of potentially long-term (100+ years), low-probability high-impact events (e.g., system collapse of boreal forests) that are often overlooked when considering risks [29] that may be beyond our contemporary frame of reference. There is growing evidence that CC-influenced natural disturbances are major drivers of C dynamics in forest ecosystems and contribute to substantial C emissions annually [3], [15], while placing infrastructure and human dwellings at risk (a set of consequences not considered here). When the consequences (even absent economic losses) and likelihoods of forest C stocks shifting from sinks to sources are viewed together, a critical need for a cohesive approach to monitoring and managing risk emerges.

**Figure 3:**
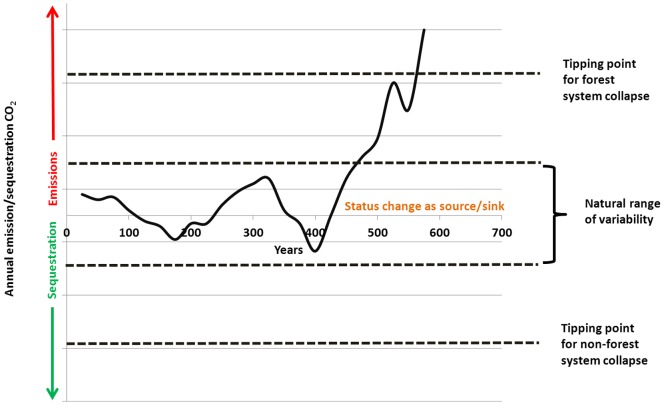
Hypothetical trend in forest ecosystem CO_2_ emissions/sequestration over a multi-century time period in the context of a natural range of variability and potential tipping points between forest ecosystems and other systems (e.g., grasslands).

As much uncertainty is associated with the fate of forest ecosystem C following natural disturbance and the potential feedbacks between forests and climate [11], [12], [30] new questions emerge. What is the likelihood that US forests will once again become net emitters of CO_2_? Is there a tipping point [31] at which CC and associated disturbances fundamentally alter forest ecosystem processes (e.g., regeneration and decay) such that the current system collapses with a concomitant large release of C? This scenario can be viewed hypothetically wherein a forest ecosystem as it proceeds through time within a natural range of variability (i.e., disturbance and subsequent recovery) regarding its C source/sink status (Fig 3). As seen in the US, forest ecosystems and their status as a sink of C partially recovered despite a transcontinental forest disturbance (i.e., 1700–1910 widespread harvest and land-use conversion) that shifted the forest's source/sink status past what might be considered a natural range of variability [7] (Fig 1). The ability of forests to recover (i.e., sequester and accumulate forest C) following disturbance has been widely documented [32]. However, there may be a tipping point where specific disturbances and/or CC push a forest ecosystem beyond the point from which it can recover [31], potentially resulting in new systems (e.g., shrub/steppe). Conversely, disturbance regimes and changing climates that are conducive to forest ecosystems could result in the invasion of non-forest systems by forest communities (Fig 3). Beyond conjecture, framing these risks with empirical observations may refine mitigation/adaptation efforts while directing future technical refinements (i.e., calculation of risk).

The empirical parameterization of the risk matrix in this study highlights numerous knowledge gaps: 1) robust measures of forest C stock change likelihood under climate change scenarios, 2) projections of forest C stocks given unforeseen socioeconomic possibilities (i.e., land-use change), and 3) appropriate social responses to global change events for which there is no contemporary climate/disturbance analog.

First, the initial metric of C stock change likelihood in this study (coefficient of variation across climatic regions) was for illustrative purposes and falls short in various situations (e.g., coarse spatial resolution of future climate projections can inadvertently reduce coefficients of variation). There is the artifact of modeled versus empirical C estimates that affects the risk matrix. Stochastic disturbance events may not be accurately reflected in the coefficients of variation as some pools are modeled as a function of stand attributes (e.g., forest type) that may not be similarly impacted as the modeled pool. Woodall et al. [33] found that modeled standing dead tree C stocks across the US may not accurately reflect the empirical variation inherent with stochastic mortality events (e.g., pine beetle mortality). If forest C inventories adopt more empirically-based assessments of C stocks (e.g., increased sample intensity or advanced remotely sensed imagery techniques) then perhaps the risk matrix would be more responsive to CC-induced disturbances. Although adequately gauging uncertainty (i.e., variation of forest C stocks) is an essential component of greenhouse gas inventories that facilitates societal policy discussions [34], there yet remains a robust method that could be employed in this study. If indeed measures of likelihood of forest C stock change are needed for society to appropriately value forest management actions (i.e., mitigation and adaptation activities) [35] then perhaps this remains the largest knowledge gap identified in this study.

Second, the size of future forest C stocks was linearly extrapolated to the year 2100 in this study which we acknowledge has a low probability of occurring [36], [37]. Although the projected trends in forest ecosystem C stocks from 2010 to 2100 provide some insight into the direction and extent of potential change at a continental scale, it does not provide the level of detail to forecast, for example, the location and extent of fluctuations in the live biomass pool following a conversion of forest lands to grasslands due to changes in precipitation cycles, wildfires, and/or tree regeneration failure (i.e., system collapse). A hurdle in using a risk framework to guide social response is how far a given pool would move within the framework after a CC-induced event (i.e., how far and in what direction the sink negative/positive arrows extend). For example, recent evidence supports suggestions that CC will result in more frequent higher-intensity storms in the future [38]. While debate still surrounds the prediction of an increase in the number of storms, there appears to be considerable agreement that CC is likely to increase storm intensity and duration [11], [13], [14], [39]. Beyond CC induced changes in forest C stock magnitude, there is perhaps the larger question regarding future socioeconomic conditions that often guide changes in forest C stocks. The economic recession of 2008 had a deleterious effect on forest and housing industries in the US [40], but assuming pre-2008 land-use trends to 2030 suggested reduction in the rate of US forest C sequestration [36]. Sharp changes in socioeconomic dynamics can have greater effect on forest C stocks than CC thus confounding technical advances to project forest ecosystem attributes under various climate scenarios.

Third, as this study only examined a limited set of societal responses to global change considered by previous studies [20], [21], alternative response categories particular to forest C management should be considered. Despite the founding of the United Nation's Intergovernmental Panel on Climate Change nearly 25 years ago followed by a bevy of research and societal response (e.g., Reducing Emissions from Deforestation and Forest Degradation), the global CO_2_ concentration continues to increase at an increasing rate [9]. Risk matrices, such as the one proposed in this study, may need to be constructed to accept evolving responses to CC such as emphasizing adaptation as opposed to mitigation. Furthermore, the cost metric of source/sink status of forest C stocks in this study may need to be changed to one of C baselines commonly used in C monitoring (e.g., 1990 baseline year) [10]. Perhaps likelihood could be defined as the likelihood of a certain percentage reduction in net C balance below a baseline projection or even historic benchmark. If adaptation is favored in lieu of mitigation activities then perhaps a cost-metric of gain/loss of forest land area or tree regeneration stocking could be used. If uncertainty of a forest C risk matrix (both future stock magnitude and likelihood of change) remains relatively high then it should follow that society's response and cost metrics should be flexible within a risk matrix.

## Conclusions

Overall, the risk framework promulgated in this study not only offers an approach to identifying knowledge gaps associated with forest C dynamics in the context of global change, but also a generalized path to prioritizing research and monitoring, given risks associated with future CC. The risk of any particular forest C stock becoming a net atmospheric emission is related to its particular stock attributes (e.g., stock size and pool) and surrounding climate (e.g., equatorial versus temperate). These factors form a complex matrix that CC may inherently alter in unforeseen ways. The empirical parameterization of such a risk matrix highlights numerous major knowledge gaps: 1) robust measures of forest C stock change likelihood under climate change scenarios, 2) projections of forest C stocks given unforeseen socioeconomic possibilities (i.e., land-use change), and 3) appropriate social responses to global change events for which there is no climate/disturbance analog. Given the finite ability of our society to alter future climate trajectories and disturbances associated with global change, using a risk framework to address the greatest risks to forest C stocks may provide one path to future forest sustainability. Despite the qualitative nature and research gaps within the forest C stock risk framework, this approach provides a conceptual way forward for identifying priority research needs for mitigating or adapting to potential CC-induced events.

## References

[1] Woodall CW, Skog K, Smith JE, Perry CH (2011) Criterion 5: Climate change and global carbon cycles. In National Report on Sustainable Forests – 2010. Editors: Robertson G, Gaulke P, McWilliams R, LaPlante S, Guldin R. FS-979. Washington DC: U.S. Department of Agriculture, Forest Service.

[2] Davidson EA, Janssens IA (2006) Temperature sensitivity of soil carbon decomposition and feedbacks to climate change. Nature 440: 165–173. 16525463 10.1038/nature04514

[3] Kurz WA, Stinson G, Rampley GJ, Dymond CC, Neilson ET (2008) Risk of natural disturbances makes future contribution of Canada's forests to the global carbon cycle highly uncertain. Proc Natl Acad Sci U S A 105: 1551–1555. 18230736 10.1073/pnas.0708133105 PMC2234182

[4] Melillo JM, Butler SM, Johnson JE, Mohan JE, Lux H, et al (2011) Soil warming, carbon-nitrogen interactions and forest carbon budgets. Proc Natl Acad Sci USA 108: 9508–9512. 21606374 10.1073/pnas.1018189108 PMC3111267

[5] Anderegg WRL, Berry JA, Smith DD, Sperry JS, Anderegg LDL, et al (2012) The roles of hydraulics and carbon stress in a widespread climate-induced forest die-off. Proc Natl Acad Sci USA 109: 233–237. 22167807 10.1073/pnas.1107891109 PMC3252909

[6] Houghton RA, Hackler JL, Lawrence KT (1999) The U.S. Carbon Budget: Contributions from Land-Use Change. Science 5427: 574–578. 10.1126/science.285.5427.574 10417385

[7] Birdsey R, Pregitzer K, Lucier A (2006) Forest carbon management in the United States: 1600–2100. J Environ Qual 35: 1461–1469. 16825466 10.2134/jeq2005.0162

[8] Etheridge DM, Steele LP, Langenfelds RL, Francey RJ, Barnola JM, et al. (1998) Historical CO_2_ records from the Law Dome DE08, DE08-2, and DSS ice cores. 1998. In Trends, "A compendium of data on global change," Carbon Dioxide Information Analysis Center, Oak Ridge National Laboratory, Oak Ridge, TN. http://cdiac.esd.ornl.gov/trends/co2/lawdome.html.

[9] ESRL (2012) Trends in atmospheric carbon dioxide. U.S. Department of Commerce, National Oceanic and Atmospheric Administration, Earth System Research Laboratory, Global Monitoring Division. http://www.esrl.noaa.gov/gmd/ccgg/trends/.

[10] EPA (2011) Inventory of U.S. greenhouse gas emissions and sinks: 1990–2009. Chapter 7. Land use, land-use change, and forestry. Annex 3.12. Methodology for estimating net carbon stock changes in forest land remaining forest lands. Washington, DC: U.S. Environmental Protection Agency. #430-R-11-005.

[11] Dale VH, Joyce LA, McNulty S, Neilson RP, Ayres MP, et al (2001) Climate change and forest disturbance. BioScience 51: 723–734.

[12] Running SW (2008) Ecosystem disturbance, carbon, and climate. Science 321: 652–653. 18669853 10.1126/science.1159607

[13] Turner MG (2010) Disturbance and landscape dynamics in a changing world. Ecology 91: 2833–2849. 21058545 10.1890/10-0097.1

[14] Seidl R, Schelhaas MJ, Lexer MJ (2011) Unraveling the drivers of intensifying forest disturbance regimes in Europe. Glob Chang Biol 17: 2842–2852.

[15] Zeng HC, Chambers JQ, Negron-Juarez RI, Hurtt GC, Baker DB, et al (2009) Impacts of tropical cyclones on US forest tree mortality and carbon flux from 1851to 2000. Proc Natl Acad Sci USA 106: 7888–7892. 19416842 10.1073/pnas.0808914106 PMC2683102

[16] Pan Y, Birdsey RA, Fang J, Houghton R, Kauppi PE, et al (2011) A large and persistent carbon sink in the world's forests. Science 333: 988–993. 21764754 10.1126/science.1201609

[17] Reich PB (2011) Taking stock of forest carbon. Nat Clim Chang 1: 346–347.

[18] Raiffa H, Schlaiffer R (2000) Applied statistical decision theory. Wiley Classics, New York.

[19] Turner II BL, Kasperson RE, Matson PA, McCarthy JJ, Corell RW, et al (2003) A framework for vulnerability analysis in sustainability science. Proc Natl Acad Sci USA 100: 8074–8079. 12792023 10.1073/pnas.1231335100 PMC166184

[20] Iverson LR, Matthews SN, Prasad AM, Peters MP, Yohe G (2012) Development of risk matrices for evaluating climatic change responses of forested habitats. Clim Change 114: 231–243.

[21] Yohe G (2010) Risk assessment and risk management for infrastructure planning and investment. Bridge 40: 14–21.

[22] Yohe G, Leichenko R (2010) Adopting a risk-based approach. Ann N Y Acad Sci 1196: 29–40. 20545647 10.1111/j.1749-6632.2009.05310.x

[23] Bechtold WA, Patterson PJ (2005) The enhanced Forest Inventory and Analysis program—national sampling design and estimation procedures. USDA For. Serv. Gen. Tech. Rep. SRS-80. 85 p.

[24] Woodall CW, Heath LS, Domke GM, Nichols M, Oswalt CM (2011) Methods and models for estimating volume, biomass, and C for forest trees in the U.S's national inventory, 2010. USDA For. Serv. Gen Tech. Rep. NRS-88. 30 p.

[25] Rubel F, Kottek M (2010) Observed and projected climate shifts 1901–2100 depicted by world maps of the Köppen-Geiger climate classification. Meteorol. Z. 19: 135–141.

[26] Pimm SL, Jones HL, Diamond J (1988) On the risk of extinction. Am Nat. 132: 757–785.

[27] Brief RP, Owen J (1969) A note on earnings risk and the coefficient of variation. Finance 24: 901–904.

[28] Grosse G, Harden J, Turetsky M, McGuire AD, Camill P, et al (2011) Vulnerability of high-latitude soil organic carbon in North America to disturbance. J Geophys Res 116: G00K06.

[29] Weitzman ML (2011) Fat-tailed uncertainty in the economics of catastrophic climate change. Rev Environ Econ Policy 5: 275–292.

[30] O'Halloran TL, Law BE, Goulden ML, Wang Z, Barr JG, et al (2012) Radiative forcing of natural forest disturbances. Glob Chang Biol 18: 555–565.

[31] Walker G (2006) The tipping point of the iceberg: Could climate change run away with itself? Nature 441: 802–805. 16778859 10.1038/441802a

[32] Amiro BD, Barr AG, Barr JG, Black TA, Bracho R, et al (2010) Ecosystem carbon dioxide fluxes after disturbance in forests of North America. J Geophys Res 115: G00K02.

[33] Woodall CW, Domke GM, MacFarlane DW, Oswalt CM (2012) Comparing field- and model-based standing dead tree carbon stock estimates across forests of the United States. Forestry 85: 125–133.

[34] Jonas M, Marland G, Winiwarter W, White T, Naborski Z, et al (2010) Benefits of dealing with uncertainty in greenhouse gas inventories: introduction. Clim Change 103: 3–18.

[35] Hurteau MD, Hungate BA, Koch GW (2009) Accounting for risk in valuing forest carbon offsets. Carbon Balance Manag 4: 1. 19149889 10.1186/1750-0680-4-1 PMC2651132

[36] U.S. Department of Agriculture, Forest Service (2012) Future of America's forest and rangelands: Forest Service 2010 Resources Planning Act Assessment. Gen. Tech. Rep. WO-87. Washington, DC. 198 p.

[37] Zhao M, Running SW (2010) Drought-induced reduction in global terrestrial net primary production from 2000 through 2009. Science 329: 940–943. 20724633 10.1126/science.1192666

[38] Elsner JB (2006) Evidence in support of the climate change–Atlantic hurricane hypothesis. J Geophys Res 33: L16705.

[39] Knutson TR, McBride JL, Chan J, Emanuel K, Holland G, et al (2010) Tropical cyclones and climate change. Nat Geosci 3: 147–163.

[40] Woodall CW, Ince PJ, Skog KE, Aguilar FX, Keegan CE, et al (2012) An overview of the forest products sector downturn in the US. For Prod J 61: 595–603.

